# Factors Associated with Health-Related Quality of Life in Community-Dwelling Older Adults: A Multinomial Logistic Analysis

**DOI:** 10.3390/jcm8111810

**Published:** 2019-11-01

**Authors:** Encarnación Blanco-Reina, Jenifer Valdellós, Ricardo Ocaña-Riola, María Rosa García-Merino, Lorena Aguilar-Cano, Gabriel Ariza-Zafra, Inmaculada Bellido-Estévez

**Affiliations:** 1 Pharmacology and Therapeutics Department, School of Medicine, Instituto de Investigación Biomédica de Málaga-IBIMA, University of Málaga, 29016 Málaga, Spain; ibellido@uma.es; 2 Health District of Málaga-Guadalhorce, 29009 Málaga, Spain; jenny_dok7@hotmail.com; 3 Escuela Andaluza de Salud Pública, 18011 Granada, Spain; ricardo.ocana.easp@juntadeandalucia.es; 4 Instituto de Investigación Biosanitaria ibs.GRANADA, 18012 Granada, Spain; 5 Health District of Córdoba Sur, 14940 Córdoba, Spain; rosaballet@yahoo.es; 6 Physical Medicine and Rehabilitation Department, Hospital Regional Universitario, 29010 Málaga, Spain; loagca2011@hotmail.com; 7 Geriatrics Department, Complejo Hospitalario Universitario, 02006 Albacete, Spain; gariza@sescam.jccm.es

**Keywords:** health-related quality of life, older adults, frailty, medication, primary care

## Abstract

The main aim of this study was to determine the association of various clinical, functional and pharmacological factors with the physical (PCS) and mental (MCS) summary components of the health-related quality of life (HRQoL) of community-dwelling older adults. Design: Cross-sectional study. Patients and setting: Sample of 573 persons aged over 65 years, recruited at 12 primary healthcare centres in Málaga, Spain. Sociodemographic, clinical, functional, and comprehensive drug therapy data were collected. The main outcome was HRQoL assessed on the basis of the SF-12 questionnaire. A multinomial logistic regression model was constructed to study the relationship between independent variables and the HRQoL variable, divided into intervals. The average self-perceived HRQoL score was 43.2 (± 11.02) for the PCS and 48.5 (± 11.04) for the MCS. The factors associated with a poorer PCS were dependence for the instrumental activities of daily living (IADL), higher body mass index (BMI), number of medications, and presence of osteoarticular pathology. Female gender and the presence of a psychopathological disorder were associated with worse scores for the MCS. The condition that was most strongly associated with a poorer HRQoL (in both components, PCS and MCS) was that of frailty (odds ratio (OR) = 37.42, 95% confidence interval (CI) = 8.96–156.22, and OR = 20.95, 95% CI = 7.55–58.17, respectively). It is important to identify the determinant factors of a diminished HRQoL, especially if they are preventable or modifiable.

## 1. Introduction

The aging of the population is a global phenomenon that is producing dramatic sociodemographic transformations. In this respect, a recent study modelled life expectancy, all-cause mortality and cause of death forecasts for 250 causes of death from 2016 to 2040 in 195 countries and territories. For 2040, Japan, Singapore, Spain and Switzerland were forecast to have an average life expectancy exceeding 85 years for both genders, and another 59 countries, including China, were projected to surpass a life expectancy of 80 years. According to these forecasts, Spain will then have the greatest life expectancy in the world (85.8 years) [1]. This pattern of aging poses a major challenge to health and social assistance services, as older people present more chronic conditions and generate higher per capita healthcare costs. Multimorbidity is strongly associated with adverse health outcomes, such as disability, dependence, mortality, increased need for health and social services and polymedication, and diminished health-related quality of life (HRQoL) [2].

Increased longevity should not be achieved at the expense of quality of life. A poor HRQoL has been associated with reduced activities of daily living, a higher frequency of hospitalisation and increased mortality [3,4]. Therefore, enhancing HRQoL should be a major consideration in the design and implementation of health care for older persons [5]. In addition, assigning a greater importance to the quality of life, in preference to disease-based outcomes, is consistent with the opinions expressed by such persons themselves [6,7]. As a consequence of the interest being generated by this topic, patient-perceived quality of life is now widely used as a measure of health care in clinical research and in health economics assessments [8].

HRQoL has been defined as “the subjective perception influenced by the current health status of the ability to perform activities important for the person” [9,10]. In response to heightened interest in assessing HRQoL and in determining the effectiveness of health care interventions in older patients, a range of instruments—both generic and specific—have been developed for these purposes.

Many factors can influence the HRQoL of older adults, including health status, social engagement and cognitive function [11]. In this area, studies have been undertaken to analyse the association between factors (such as female sex, age, functional impairment and comorbidities) and HRQoL, and in recent years, the role of medication as a determinant of overall health outcomes has emerged as an area of great interest. Various aspects of treatment regimens call for special attention, because they may have a negative influence on the HRQoL of older persons, such as polymedication, and potentially inappropriate or harmful medication [12,13,14,15,16,17]. It is important to recall that the benefits of medication should always outweigh the potential harm, and therefore individual circumstances should be taken into account. In recent studies, frailty, too, has been highlighted as a possible determinant of worsened quality of life [18,19,20,21,22]. Frailty is defined as an abnormal health state characterised by the loss of biological reserves, related to the aging process [23]. Therefore, in providing care for persons with frailty, special emphasis should be placed on quality of life considerations.

Evaluation of HRQoL can help clinicians to determine the needs of older patients and thus optimise their decision making. Therefore, we believe it is important to explore factors influencing HRQoL in this population in order to identify suitable intervention strategies. In this respect, we note that many previous studies have not addressed all the factors that might have a significant impact on HRQoL, and in some cases the results presented are contradictory. For all of these reasons, we consider it interesting to investigate HRQoL in older adults, resident in one of the countries where life expectancy is highest, to assess clinical, functional and pharmacological aspects of the question. In this study, we hypothesise that there are multiple predictive factors of a poor HRQoL and that they may affect its physical and mental components in different ways and to different degrees. Accordingly, our aim was to determine levels of HRQoL among community-dwelling older people and to analyse the factors associated with a poor HRQoL.

## 2. Patients and Methods

### 2.1. Study Design, Setting and Participants

In this cross-sectional investigation, the study population was composed of 89,615 community-dwelling residents aged 65 years or more, living in Málaga, Spain. Assuming a standard deviation for HRQoL of 11.0 [24], an absolute precision of δ = 0.9 and a level of confidence of 1−α = 0.95, we calculated that the minimum sample size needed to estimate the average physical and mental HRQoL was 570. Finally, the total sample was composed of 573 persons. These patients were recruited from twelve primary care centres, by stratified random sampling designed to obtain a representative sample of the population, allocating the population in proportion to the size of each healthcare centre. Participants were selected randomly within each healthcare centre from a general list of healthcare cards issued by the Spanish National Health Service. The inclusion criteria were people 65 years of age or older, included in the database of healthcare cards, belonging to the outpatient setting (not institutionalized), and giving their informed consent to participate in the study (people who did not give consent were excluded).

### 2.2. Data Collection and Measures

To obtain the study data for analysis, patients were interviewed using a structured questionnaire. Further data were obtained from medication packaging and digital medical records. The questionnaire was used to obtain detailed information on the patients’ regular drug use, together with clinical, functional and sociodemographic data. Clinical diagnoses were examined, and the number of chronic conditions presented by each participant was determined. Patients’ independence in performing instrumental activities of daily living (IADL) was assessed using the Lawton scale [25]. In addition, the body mass index was determined for all patients, and frailty was assessed according to Fried’s criteria (as robust, pre-frail or frail) [26].

Medication assessment. Data were obtained for the medication prescribed (indication, dosage and duration of treatments during the last three months or more). The presence of polymedication was considered, defining this as the regular use of five or more medications, as was that of potentially inappropriate medication (PIM), according to the STOPP v2 criteria (Screening Tool of Older Person’s Potentially Inappropriate Prescriptions, version 2) [27]. The latter variable was operationalised as the percentage of patients receiving at least one PIM.

Quality of life assessment. The main study outcome was HRQoL assessed by the SF-12 questionnaire, a widely used generic instrument. The SF-12 is an abbreviated version of the Short Form-36 Health Survey (SF-36), in which a subset of 12 items/questions are used to derive summary scores for physical health (PCS score) and mental health (MCS score) [28]. The response options form Likert-type scales that assess the intensity and/or frequency of people’s health status. The final score obtained can be between 0 and 100, where lower scores indicate worse, and higher scores better HRQoL. Using only one-third of the SF-36 items, the SF-12 reproduces the two summary scores originally developed for the SF-36 with remarkable accuracy, but more quickly and requiring less effort from the respondent [29]. The SF-12 has been validated for use in the USA, the UK, Spain and many other European countries [24].

### 2.3. Statistical Analysis

Exploratory data analysis and frequency tables were used to describe the study variables. Using the standardised scores of the SF-12 questionnaire for the Spanish population [24], the HRQoL score of each participant was assigned to one of the following intervals, taking into account the population reference group according to age and sex:

- Very low: QoL ≤ 20th percentile of the Spanish population corresponding to their age group and sex;
- Low: QoL > 20th percentile and ≤50th percentile of the Spanish population corresponding to their age group and sex;
- High: QoL > 50th percentile and ≤80th percentile of the Spanish population corresponding to their age group and sex;
- Very high: QoL > 80th percentile of the Spanish population corresponding to their age group and sex.

A multinomial logistic regression model was used to study the relationship between the independent variables and the HRQoL variable, grouped into the corresponding intervals [30]. A 5% significance level was assumed to indicate statistical significance. Statistical data analysis was performed using SPSS version 23.0 (IBM SPSS Statistics, Armonk, NY, USA).

### 2.4. Ethical Considerations

This study was conducted in accordance with the provisions of the 1975 Declaration of Helsinki, revised in 2013. The Málaga Clinical Research Ethics Committee approved the study (PI-0234-14), and informed consent was obtained from all patients prior to their inclusion.

## 3. Results

### 3.1. Characteristics of the Study Population

The study sample was composed of 573 patients, with a mean age of 73.1 years (standard deviation 5.5, range 65–104) and of whom 57.2% were female. Most lived with their partner (62.4%) or family (16.5%), but 21.1% lived alone. On average, each patient presented 7.8 chronic conditions (standard deviation 3.3, range 0–20). The most prevalent diagnoses were bone and joint disorders (mainly osteoarthritis of the knee, hip, hand and shoulder) (75.2%), hypertension (70.5%) and dyslipidaemia (51.6%). Most of the patients presented with overweight (40.9%) or obesity (45.7%) and their mean body mass index was 30.2 (standard deviation 5.1, range 17–54.5). Half were independently capable of performing IADL, and the mean score on the Lawton scale was 6.6 (standard deviation 1.8, range 0–8). Frailty was present in 137 patients (23.9%; 95% confidence interval (CI) = 20.5–27.5), according to Fried’s criteria. The main characteristics of the study population are detailed in Table 1.

The prevalence of polymedication was 68% (95% CI = 64.1–71.7), and on average each patient consumed 6.8 drugs (standard deviation 4.0; range 0–23). The most widely prescribed drugs were omeprazole and acetaminophen, followed by aspirin, simvastatin, metformin, metamizole, enalapril and bromazepam. The use of potentially inappropriate medication, according to the STOPP v2 criteria, was identified in 66.8% of patients (95% CI = 62.9–70.6). The number of PIMs per patient ranged from 0–10 (mean 2.1, standard deviation 2.2). The most frequent PIMs detected were benzodiazepines (61% of all PIMs).

### 3.2. Assessment of HRQoL and Analysis of Related Factors

The patients’ perceptions of their HRQoL produced an average score of 43.2 (standard deviation 11.02, range 16.2–65.4) for the physical component summary (PCS) and a somewhat higher one, 48.5 (standard deviation 11.04, range 14.1–66.6), for the mental component (MCS). Males obtained higher scores than females in both the PCS and the MCS, with average values of 45.91 vs. 41.27 and 51.95 vs. 45.91, respectively. Table 2 shows the distribution within the global sample among the categories of perceived HRQoL (very low, low, high and very high). A notable feature of this distribution is that a higher proportion of patients perceived their HRQoL as very high in the mental component than in the physical one.

To further examine the impact of the independent variables on the HRQoL categories, a multinomial logistic regression analysis was performed (Table 3, Table 4 and Table 5). The factors related to having high vs. very high HRQoL (P_50_–P_80_ and ≥ P_80_, respectively) were the level of dependence for IADL in the PCS, and BMI, respiratory disease and frailty in the MCS (Table 3). The presence of a low HRQoL (P_20_–P_50_) with respect to a very high score for the PCS was associated with the level of dependence for IADL, with accompanied living, with the presence of osteoarticular pathology and with frailty (Table 4). The odds of these older persons having a low HRQoL decrease by 30% for each additional point of independence on the Lawton scale (odds ratio (OR) = 0.70, 95% CI = 0.55–0.88). However, they double for those who do not live alone (OR = 2.13, 95% CI = 1.07–4.27), are 2.5 times greater for those with osteoarticular pathology compared to those without it (OR = 2.57, 95% CI = 1.35–4.85) and are seven times greater for those who are frail (OR = 7.43, 95% CI = 2.13–25.82) compared to those who are robust. In the case of the MCS, the presence of frailty was associated with a three times greater perception of low HRQoL (OR = 3.2, 95% CI = 1.21–8.46).

A very low perception of HRQoL (≤P_20_) in the PCS was also related to the level of dependence for IADL (OR = 0.62, 95% CI = 0.48–0.79), with the presence of osteoarticular pathology (OR = 4.38, 95% CI = 1.98–9.70) and with the states of prefrailty (OR = 4.19, 95% CI = 1.61–10.86) and frailty (OR = 37.42, 95% CI = 8.96–156.22). In addition, the odds of a very low HRQoL increase by 15% for each additional drug in the treatment regimen (OR = 1.15, 95% CI = 1.02–1.30) and by 8% for each unit increase in BMI (OR = 1.08, 95% CI = 1.01–1.15). However, age was inversely related to HRQoL. Thus, all other variables being equal, for each additional year of life, the odds of the patient perceiving a very low HRQoL decreased by 8% (Table 5). A similar pattern for the age effect was observed with respect to the mental component of HRQoL; thus, each additional year of life decreased the odds of a very low HRQoL by 7%. In the MCS, too, the states of prefrailty and frailty were associated with a very low HRQoL. In this mental component, and with all other variables being equal, the female patients were 88% more likely than the males to have a very low HRQoL. Finally, the presence of a psychopathology (usually anxiety and/or depression) was related to a poorer HRQoL; the presence of any such disorder quadrupled the odds of the patient perceiving a very low HRQoL (OR = 4.69, 95% CI = 2.60–8.47).

## 4. Discussion

Overall, our results for perceived HRQoL among community-dwelling older patients in Malaga (southern Spain) are consistent with those reported in similar studies conducted in other regions of Spain [31,32] and elsewhere in Europe [33]. With respect to the components of physical and mental health, the average PCS score (43.2) is slightly higher than that obtained in previous research [31,32,33,34], while the MCS (48.5) is somewhat lower than the average value for six European countries (54.3), also obtained using the SF-12 [33]. This lower score in the MCS could be due to cultural differences, and/or the non-negligible prevalence of psychopathological disorders (mainly anxiety and/or depression) in our region (these pathologies affect 36% of the older population). Regarding the influence of age on HRQoL, previous studies have produced conflicting results [32,33,34,35]. Our investigation revealed an inverse relationship between advanced age and the odds of a very low HRQoL. Therefore, other questions such as comorbidities, frailty, dependence and other possible confounders being equal, as the patient ages there is a lower probability of him/her perceiving a very low HRQoL, and this is true for both the physical and the mental components. These findings might be explained in terms of a better psychological adaptation to aging and perhaps to the fact that with age comes not only wisdom, but also the attribution of greater meaning or value to life. As concerns the influence of gender, previous studies have reported a poorer HRQoL among women than among men [31,32,33,34,36,37], and our own results corroborate this difference. Thus, women obtained poorer scores in both components of HRQoL, although the association was only significant for the mental component of a very low quality of life. In the physical component, female gender is probably not a determinant factor of a poorer HRQoL due to the adjustment made for confounding variables such as frailty or osteoarticular pathology, both of which are more prevalent among women.

One of the most consistent predictors of a poor quality of life is the level of dependence for IADL. According to the multinomial logistic regression model, with a higher score on the Lawton scale, i.e., with greater independence, the possibility of a poorer HRQoL decreased significantly (in all the categories considered: high, low and very low, with respect to very high) always with respect to the physical component. It seems logical that the higher the degree of independence among older persons, in activities such as handling economic affairs, using the telephone or managing different forms of transport, the better the quality of life they will perceive. According to a recent study, functional dependence, together with the presence of depressive symptoms, would be an important factor mediating the well-known association between multimorbidity and a poor quality of life [38]. In this line, too, it has been shown that disability is one of the most significant conditions worsening the HRQoL [39]. Older adults who live in cohabitation are twice as likely to have a low physical HRQoL than are those living alone. This finding may be related to the level of functional dependence presented, but further study is needed to confirm this possibility.

Regarding medication, the number of drugs prescribed was positively associated with a very low HRQoL in the PCS. This finding is consistent with previous research, in which polymedication has been identified as a determinant factor of a poorer quality of life [12,13,14,40]. In our opinion, this association may be related to the side effects produced by the joint presence of several drugs within the patient. On the other hand, we found no evidence of any association between having at least one PIM and the perception of a poorer HRQoL. We speculate that such a relationship might not have been demonstrated because the STOPP v2 criteria contain a large number of items, of varying clinical significance, and therefore the impact produced on HRQoL by a single PIM might be slight. In other words, the sensitivity of this means of measuring the risk might be insufficient. Other authors, too, have failed to observe any significant association between the presence of a PIM and HRQoL, whether using the Beers [16] or the STOPP v2 criteria [41]. On the other hand, other previous studies do suggest that the prescription of drugs with a high anticholinergic load may be associated with a diminished quality of life [15,16,17,42].

Among this population, the most prevalent pathologies observed were osteoarthritis and hypertension. This is a common profile and similar to that reported in previous research in this field [39]. Our assessment of the impact on HRQoL of different conditions shows that physical disorders mostly affected the “physical” HRQoL while mental disorders mainly affected the “mental” aspect—as is only logical. The presence of bone and joint disease was significantly associated with a low or very low HRQoL, which corroborates previous findings in which this diagnosis was associated both with disability and with a diminished HRQoL [39]. We believe this relationship may be explained by the chronic pain which often accompanies these diseases, as well as a degree of physical limitation that is usually provoked. This association contrasts with its absence for other diagnoses, perhaps with a worse prognosis but of a more ‘silent’ nature, such as hypertension and diabetes mellitus. On the other hand, the presence of a psychopathology in an older person raises the possibility of his/her perceiving a very low HRQoL, in the mental component, by 400%, as has also been indicated in previous studies [12,31,32,43,44]. It would seem that the distress generated by these diseases has a negative impact on emotional regulation, motivation and other components of subjective perceptions of health and well-being. According to other researchers, and taking into account the significant impact on the patient’s quality of life, we believe more screening for depression and anxiety among older populations should be performed, because these conditions tend to be under-diagnosed and also because a more active approach to this question would promote healthy aging [31]. In our sample, the BMI values observed presented two interesting aspects: on the one hand, at the mental level, a higher BMI was associated with a better HRQoL, but for the physical component it was significantly associated with a very low quality of life. It seems clear that overweight and obese patients perceive a poorer physical health [40,45,46,47]. This is corroborated by the fact that interventions aimed at achieving weight loss have been shown to improve the physical quality of life [48].

It is interesting to note that the factor most strongly associated with a diminished HRQoL was that of frailty, which severely affected both the MCS and the PCS, but especially the latter. The odds of a very low physical quality of life were 37 times greater among frail older persons than among those who were robust, while the MCS reflected 20 times greater odds of their presenting a poor mental quality of life. Previous studies, too, have reported this association [18,19,20,21,49], which was reinforced in a recent systematic review and meta-analysis that described it as clear and often substantial [22]. Although the growing numbers of frail older people pose a real challenge to health systems around the world, this state of pre-discapacity can in fact be prevented and treated. Furthermore, in our study sample, the pre-frail persons also perceived a poorer quality of life than those who were relatively robust. Accordingly, we believe it necessary to design and incorporate care programmes specifically adapted to this emerging population of frail and pre-frail patients, to help them age with a better quality of life.

In our opinion, the type of study described in this paper is useful for identifying the characteristics and clinical conditions that are associated with a poorer HRQoL, with particular attention to those factors that may be preventable or treatable. As improving the quality of life and well-being of older persons is a priority objective, it is of major importance to extend our knowledge of these questions.

The strengths of our study lie in the analysis made of a representative sample of healthcare centres, the global approach taken, and the great variety of clinical, functional and treatment data compiled. We acknowledge that selecting a sample population from a single region or country may result in a certain lack of external validity. Nevertheless, the sample examined in this study may be representative of the population of older adults in the ambulatory setting, which is where the largest number of such patients are to be found. Another limitation to our study is its cross-sectional design, which does not allow causal relationships to be established, although it can detect factors related to HRQoL.

With regard to the analysis performed, since HRQoL is a quantitative variable, a multivariate linear regression model might, in principle, be considered appropriate to identify the factors related to it. However, in the present case the linear model considered did not meet the conditions of homoscedasticity and normality of the residuals necessary for the correct estimation of the study parameters. Previous statistical research has reached similar conclusions, i.e., that HRQoL cannot be analysed using linear regression models [50]. Neither nonlinear modelling, nor generalised least squares nor other more complex statistical techniques overcame the problem of achieving an adequate fit, and therefore we adopted the solution of treating QoL as a qualitative variable and using a multinomial logistic regression model. This approach provided a good fit to the data considered.

## 5. Conclusions

In conclusion, many factors may be predictive of a poor HRQoL and they affect its physical and mental components in different ways. For the PCS, the associated factors were dependence for IADL, a higher BMI, the number of medications and the presence of osteoarticular pathology. The main factors associated with a lower MCS score were female gender and the presence of a psychopathological disorder. Some factors may be preventable or modifiable, and so recognising them and optimising the response made are crucial to the priority objective of enhancing the quality of life among older people. Clearly and consistently, the factor that was most strongly associated with a poorer overall HRQoL was the state of frailty (and also, albeit to a lesser extent, that of pre-frailty). Frailty is a dynamic syndrome, in which transitions are possible between the states of normality, pre-frailty and frailty. Accordingly, detecting frailty and addressing it in a suitable way are questions of major importance.

## Figures and Tables

**Table 1:** Characteristics of study population (*n* = 573).

**Quantitative Variables**	**Mean**	**Standard Deviation**
Age (years)	73.1	5.5
Lawton (IADL)	6.6	1.8
BMI (Kg/m^2^)	30.2	5.1
Number of comorbidities	7.8	3.3
Number of drugs per patient	6.8	4.0
Number of PIMs per patient	2.1	2.2
**Qualitative Variables**	**Subjects**	**Percentage**
**Gender**		
Male	245	42.8
Female	328	57.2
**Living Arrangements**		
Living alone	121	21.1
Accompanied living	452	78.9
**Frailty**		
Robust	124	21.6
Pre-frail	312	54.5
Frail	137	23.9
**Most Frequent Comorbidities**		
Bone and joint disorders	431	75.2
Hypertension	404	70.5
Dyslipidaemia	292	51.0
Insomnia	254	44.3
Gastrointestinal disease	241	42.0
Peripheral vascular disease	227	39.6
Psychopathology	207	36.1
Diabetes mellitus	172	30.0
Heart disease	139	24.3
Respiratory Disease	123	21.5
**Polymedication**	390	68.0
**PIM prevalence**	383	66.8

IADL: Instrumental Activities of Daily Living; BMI: Body Mass Index; PIM: Potentially Inappropriate Medication (according to STOPP v2 criteria).

**Table 2:** HRQoL assessment according to SF-12 questionnaire.

HRQoL Component	HRQoL Categories, *n* (%)			
Summary Score	Very low (≤P_20_)	Low (P_20_–P_50_)	High (P_50_–P_80_)	Very high (≥P_80_)
PCS	137 (23.9%)	170 (29.7%)	150 (26.2%)	116 (20.2%)
MCS	143 (25.0%)	117 (20.4%)	125 (21.8%)	188 (32.8%)

PCS: Physical Component Summary; MCS: Mental Component Summary; P: Percentile.

**Table 3:** Factors related to HRQoL. Multinomial logistic regression for High HRQoL (with respect to Very High HRQoL) for physical and mental health components (PCS-MCS).

Independent Variable	PCS OR (95% CI)	MCS OR (95% CI)
Age	1.02 (0.96–1.07)	1.01 (0.95–1.05)
No. of comorbidities	1.06 (0.93–1.20)	1.02 (0.91–1.14)
BMI	0.99 (0.94–1.05)	0.94 (0.89–0.99) *
Independence (IADL)	0.74 (0.58–0.94) *	1.17 (0.98–1.39)
No. of medications	1.07 (0.96–1.19)	1.00 (0.91–1.10)
No. of PIMs	1.10 (0.85–1.43)	1.08 (0.87–1.34)
Female gender (ref. male)	0.72 (0.38–1.34)	1.45 (0.81–2.59)
Living accompanied (ref. alone)	1.26 (0.67–2.38)	1.14 (0.61–2.14)
Bone and joint disease	1.40 (0.79–2.49)	0.94 (0.53–1.66)
Heart disease	1.35 (0.62–2.92)	0.81 (0.42–1.54)
Respiratory disease	0.71 (0.33–1.53)	2.05 (1.10–3.84) *
Hypertension	1.20 (0.67–2.15)	1.20 (0.67–2.14)
Diabetes mellitus	1.22 (0.63–2.37)	0.76 (0.42–1.36)
Psychopathology	1.11 (0.58–2.12)	1.65 (0.91–2.98)
Insomnia	1.18 (0.64–2.16)	1.26 (0.73–2.16)
Pre–frail (ref. robust)	0.87 (0.48–1.57)	0.95 (0.53–1.69)
Frail (ref. robust)	0.46 (0.11–1.81)	3.39 (1.35–8.51) **

PIMs: Potentially Inappropriate Medications (according to STOPP v2 criteria). * *p* < 0.05; ** *p* < 0.01.

**Table 4:** Factors related to HRQoL. Multinomial Logistic Regression for Low HRQoL (with respect to Very High HRQoL) for physical and mental health components (PCS-MCS).

Independent Variable	PCS OR (95% CI)	MCS OR (95% CI)
Age	0.97 (0.92–1.03)	0.98 (0.93–1.03)
No. of comorbidities	1.08 (0.95–1.23)	1.11 (0.98–1.24)
BMI	1.04 (0.98–1.10)	0.95 (0.90–0.99) *
Independence (IADL)	0.70 (0.55–0.88) **	0.99 (0.84–1.18)
No. of medications	1.11 (0.99–1.24)	0.98 (0.89–1.08)
No. of PIMs	0.94 (0.72–1.22)	1.12 (0.89–1.39)
Female gender (ref. male)	1.01 (0.52–1.91)	1.67 (0.93–3.00)
Living accompanied (ref. alone)	2.13 (1.07–4.27) *	0.84 (0.45–1.57)
Bone and joint disease	2.57 (1.35–4.85) **	0.96 (0.53–1.72)
Heart disease	1.19 (0.54–2.62)	0.85 (0.45–1.62)
Respiratory disease	1.32 (0.64–2.71)	1.54 (0.81–2.92)
Hypertension	1.75 (0.94–3.27)	0.73 (0.41–1.31)
Diabetes mellitus	0.96 (0.48–1.92)	0.75 (0.42–1.36)
Psychopathology	1.20 (0.62–2.30)	1.36 (0.74–2.50)
Insomnia	1.33 (0.71–2.47)	1.01 (0.58–1.74)
Pre–frail (ref. robust)	1.48 (0.77–2.87)	1.50 (0.81–2.78)
Frail (ref. robust)	7.43 (2.13–25.82) **	3.20 (1.21–8.46) **

* *p* < 0.05; ** *p* < 0.01.

**Table 5:** Factors related to HRQoL. Multinomial Logistic Regression for Very Low HRQoL (with respect to Very High HRQoL) for physical and mental health components (PCS-MCS).

Independent Variable	PCS OR (95% CI)	MCS OR (95% CI)
Age	0.92 (0.86–0.98) *	0.93 (0.88–0.98) *
No. of comorbidities	1.11 (0.96–1.28)	0.98 (0.87–1.10)
BMI	1.08 (1.01–1.15) *	0.99 (0.95–1.05)
Independence (IADL)	0.62 (0.48–0.79) ***	0.94 (0.79–1.12)
No. of medications	1.15 (1.02–1.30) *	0.99 (0.90–1.10)
No. of PIMs	0.94 (0.70–1.24)	1.08 (0.87–1.35)
Female gender (ref. male)	1.58 (0.76–3.29)	1.88 (1.01–3.49) *
Living accompanied (ref. alone)	1.63 (0.76–3.50)	0.63 (0.33–1.17)
Bone and joint disease	4.38 (1.98–9.70) **	1.14 (0.60–2.18)
Heart disease	0.87 (0.36–2.08)	0.74 (0.37–1.49)
Respiratory disease	1.01 (0.45–2.22)	0.94 (0.47–1.89)
Hypertension	1.50 (0.73–3.05)	0.93 (0.51–1.72)
Diabetes mellitus	1.11 (0.52–2.36)	0.88 (0.47–1.63)
Psychopathology	1.53 (0.75–3.11)	4.69 (2.60–8.47) ***
Insomnia	1.24 (0.62–2.46)	1.66 (0.94–2.91)
Pre-frail (ref. robust)	4.19 (1.61–10.86) **	2.92 (1.36–6.26) **
Frail (ref. robust)	37.42 (8.96–156.22) ***	20.95 (7.55–58.17) ***

* *p* < 0.05; ** *p* < 0.01; *** *p* < 0.001.
